# Cryo‐EM structure of orphan G protein‐coupled receptor GPR21

**DOI:** 10.1002/mco2.205

**Published:** 2023-01-25

**Authors:** Thian‐Sze Wong, Wei Gao, Geng Chen, Chen Qiu, Guodong He, Fang Ye, Zhangsong Wu, Zicheng Zeng, Yang Du

**Affiliations:** ^1^ Kobilka Institute of Innovative Drug Discovery, Shenzhen Key Laboratory of Steroid Drug, Discovery and Development, School of Medicine the Chinese University of Hong Kong Shenzhen Guangdong China; ^2^ School of Medicine Tsinghua University Beijing China; ^3^ Innovation Center for AI and Drug Discovery East China Normal University Shanghai China

**Keywords:** cryo‐EM, ECL2, GPCR, GPR21, orphan receptor

## Abstract

GPR21 belongs to class A orphan G protein‐coupled receptor (GPCR). The endogenous ligands for human GPR21 remain unidentified. GPR21 expression is associated with developing type 2 diabetes (T2DM), a multifactorial metabolic disease caused by pancreatic β‐cell dysfunction, decreasing insulin production, insulin resistance, and obesity. Animal studies suggested that GPR21 is a potential therapeutic target for T2DM treatment. The underlying mechanisms leading to GPR21 self‐activation remain unknown. In our co‐expression analysis, we noted that GPR21 could also form a stable complex with an unreported Gα protein subtype, Gαs. To gain further insights into the structural mechanisms of GPR21 activation, we employed cryo‐electron microscopy (cryo‐EM) and single‐particle analysis to resolve the high‐resolution structure of GPR21‐Gαs complexes. The clear electron density map of the GPR21‐Gαs provided direct evidence that GPR21 could couple to Gαs protein at physiological conditions. Thus, GPR21 might mediate previously unexplored pathways in normal or pathological conditions, which warrants further investigation. Structure‐guided mutagenesis and biochemical analysis revealed that extracellular loop 2 (ECL2) of GPR21 is essential for the receptor transducing intracellular signal via cAMP. Together, the new structure data reveal a novel signaling cascade of human GPR21 mediated by ECL2 and provide fundamental information for future structure‐based drug development.

## Graphical abstract

The high‐resolution structure of GPR21‐Gαs complexes was resolved. GPR21 ECL2 exhibited highly stable conformation and is inserted deeply into the orthosteric ligand‐binding pocket of GPR21. The immersed region of ECL2 was constrained in position via an intra‐loop salt bridge (K170^ECL2^ and D176^ECL2^). The new structure data reveal a novel signaling cascade of GPR21 mediated by ECL2.

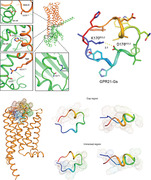

## 1 INTRODUCTION

G protein‐coupled receptors (GPCRs) represent one of the largest membrane protein families in the human genome. GPCRs are promising targets for drug development, as the receptors can transduce extracellular signals from a wide variety of ligands accurately. GPCR drug discovery is successful in the drug development pipelines.^1^ Around 34% of drugs approved by the US Food and Drug Administration (FDA) mediate their effects by modulating GPCRs activity.^2^ GPCR mediates signal transduction by activating the heterotrimeric guanine nucleotide‐binding protein (G proteins). GPCRs regulate many of the metabolic processes involved in glucose homeostasis.^3^ Agonist binding triggers hallmark conformation changes in GPCR, leading to the formation of active conformation.^4^ Thus, resolving the active conformation of GPCR is essential for designing therapeutic modulators. Receptor activation usually requires orthosteric binding of cognate ligands to the pocket formed by the N‐terminus and seven‐transmembrane (7TM) domains. GPCRs in active conformation will recruit and promote dissociation of the trimeric G protein (Gα, Gβ, and Gγ) or β‐arrestins. The intracellular G protein is a canonical signal transducer that translates extracellular stimulation to downstream effectors.^5^ In the resting state, the heterotrimeric G protein binds to the intracellular side of GPCR with GDP attached to the Gα protein subunit. Upon recruitment and stimulation by the activated receptor, GDP is replaced by GTP, resulting in GTP‐bound Gα and Gβγ subunits. The dissociated G proteins can relay signals by interacting with other cellular effectors.

Based on phylogenetic and structure similarities, GPCR can be classified into different groups: rhodopsin‐like (class A), secretin receptor‐like (class B1), adhesion receptor (class B2), metabotropic glutamate receptor (class C), fungal mating pheromone receptors (class D), cyclic AMP receptors (class E), frizzled/smoothened (class F).^6^ Class A rhodopsin family is the largest family with 13 sub‐branches. GPCR can further be grouped based on their physiological ligands. Various endogenous ligands, including neurotransmitters, nucleotides, peptides, lipids, and hormones, can activate class A GPCR. In class A GPCRs, those with no reported or well‐validated endogenous ligands are known as orphan GPCRs.^7^ Data from the Concise Guide to Pharmacology 2019/20: G protein‐coupled receptors indicate that 87 GPCRs remain classified as orphan GPCR in class A GPCR.^8^ Functional studies revealed that orphan GPCRs are essential in various physiological processes and associated with multiple pathological conditions.^9^ Orphan GPCR is regarded as a novel therapeutic target regulating signaling pathways that have not yet been explored. However, the lack of selective ligands hampers structural studies on orphan GPCR.

GPR21 belongs to class A orphan GPCR and is a novel treatment target for type 2 diabetes, primarily caused by systemic insulin resistance. Transcriptomic analysis showed that GPR21 has broad distribution and is particularly enriched in the brain, lymph node, spleen, salivary gland, and white adipose tissues.^10^ GPR21 knockout mice are resistant to diet‐induced obesity with higher glucose metabolism and insulin sensitivity. In the HEK293T cells, GPR21 is an active receptor that negatively controls insulin responsiveness.^11^ GPR21 may act via MAPK, which promotes the phosphorylation of IRS1 in the insulin signaling cascade. Dysregulation of GPR21 is observed in genetic and diet models of metabolic syndrome.^12^ Further, transcriptomic GPR21 expression in adipocytes is responsive to inflammatory stimuli.^13^ At present, the endogenous peptide ligands are yet to be identified. Leonard et al. show that synthetic inverse agonists targeting GPR21 could alter insulin‐induced glucose uptake in GPR21‐overexpressing cells.^11^ Hence, resolving the atomic structure of orphan GPR21 is essential to understanding the mechanism of action. Further, structural data of GPR21 are necessary to design and develop specific modulators to correct insulin signaling dysregulation contributed by the constitutively active GPR21.

Here, we resolve the high‐resolution structures of apo‐GPR21 using cryo‐electron microscopy (cryo‐EM) and single‐particle analysis. A previous study on HEK293T reveals that GPR21 is coupled to Gαq type G protein, which mediated its signal transduction function via MAPK.^11^ In our co‐expression analysis, we noted that GPR21 could also form a stable complex with Gαs type G protein. Structure‐guided mutagenesis and biochemical analysis revealed that the extracellular loop 2 (ECL2) of GPR21 is essential for Gαs‐coupling GPR21 to transduce cAMP signals. GPR21 ECL2 exhibited highly stable conformation and was inserted deeply into the orthosteric ligand‐binding pocket of GPR21. The unique conformation may contribute to the observed constitutive activity of GPR21 via Gαs‐signaling. Together, the new structure data reveal a novel signaling cascade of human GPR21 mediated by ECL2 and provide fundamental information for future structure‐based drug development.

## 2 RESULTS

### 2.1 The active conformation of ligand‐free GPR21

**FIGURE 1:**
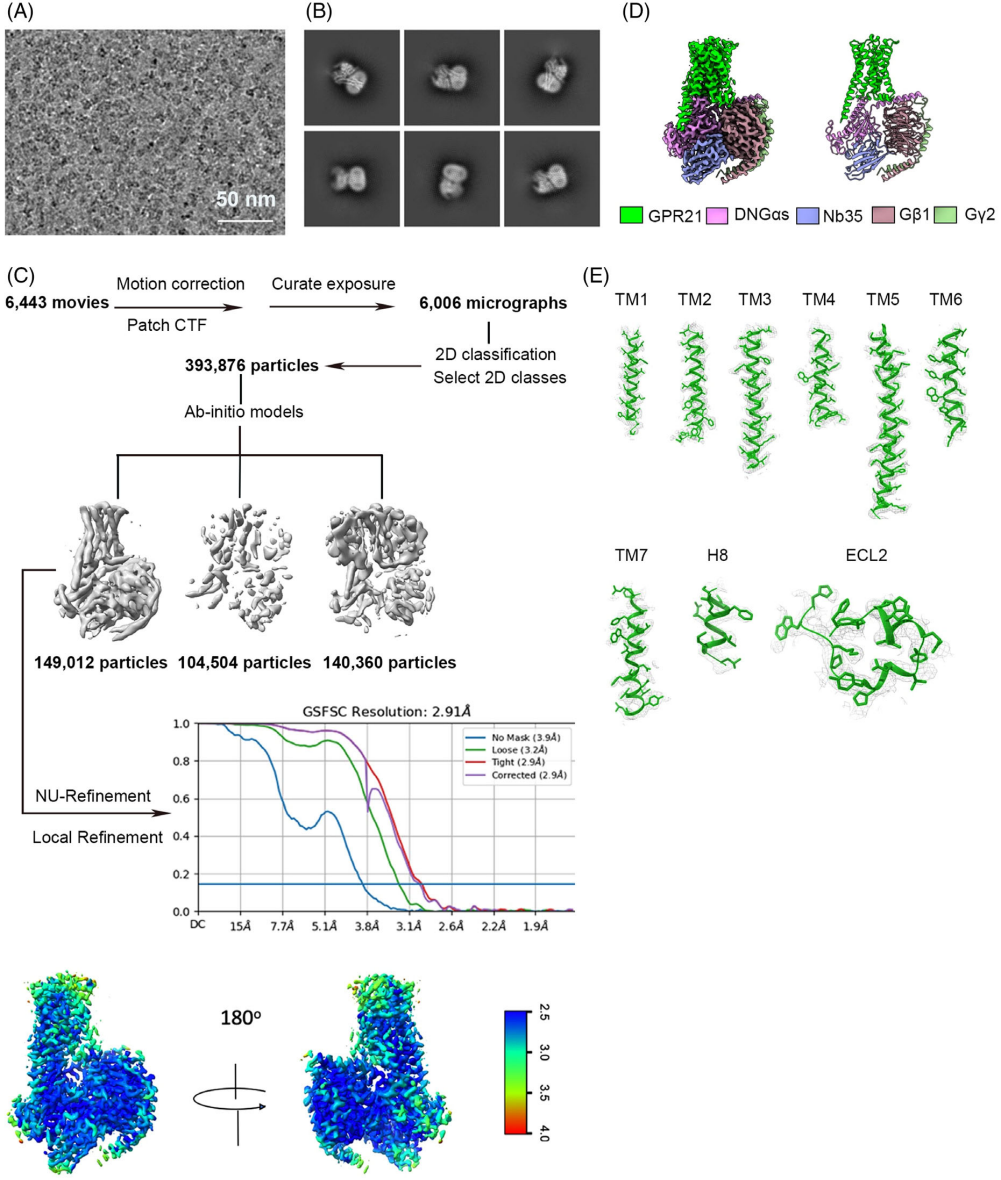
Cryo‐EM data processing of GPR21. (A) Representative micrograph of the complex particles. (B) Representative 2D images. (C) Workflow for cryo‐EM image processing. Gold standard Fourier shell correlation (FSC) curve indicates overall nominal resolution at 2.91 Å using the FSC = 0.143 criterion. (D) A cartoon representation of cryo‐EM 3D reconstruction of full‐length GPR21 (2.91 Å). GPR21 in complex with the human DNGαs, Gβ1 and Gγ2 together with G protein complex‐stabilizing nanobody Nb35. (E) Representative density maps and models for TM1‐7, H8 and ECL2 of GPR21

To obtain the pure GPR21‐G protein complexes, wild‐type GPR21 with BRIL‐fusion was expressed in *Spodoptera frugiperda* (Sf9) insect cells. To obtain the native structure of GPR21, no mutation was introduced to the expression construct for stabilization purposes. Although there is no reported ligand for GPR21, we could still purify stable GPR21‐G protein heterocomplexes. GPR12 is reported as a Gαq‐coupling GPCR. In Sf9, however, we could not purify stable GPR21‐Gαq complex (data not shown). Compared to the complex formation efficacy of GPR21 with other G protein members in Sf9, GPR21 can be stabilized by co‐expressing with Gαs. The results suggested that Gαs‐coupling may offer preferential stability to GPR21 under physiological conditions. The GPR21‐Gs‐Nb35 structure obtained by cryo‐EM analysis has a nominal global resolution of 2.91 Å (Figure 1).

### 2.2 Comparison of GPR21 and GPR52

**FIGURE 2:**
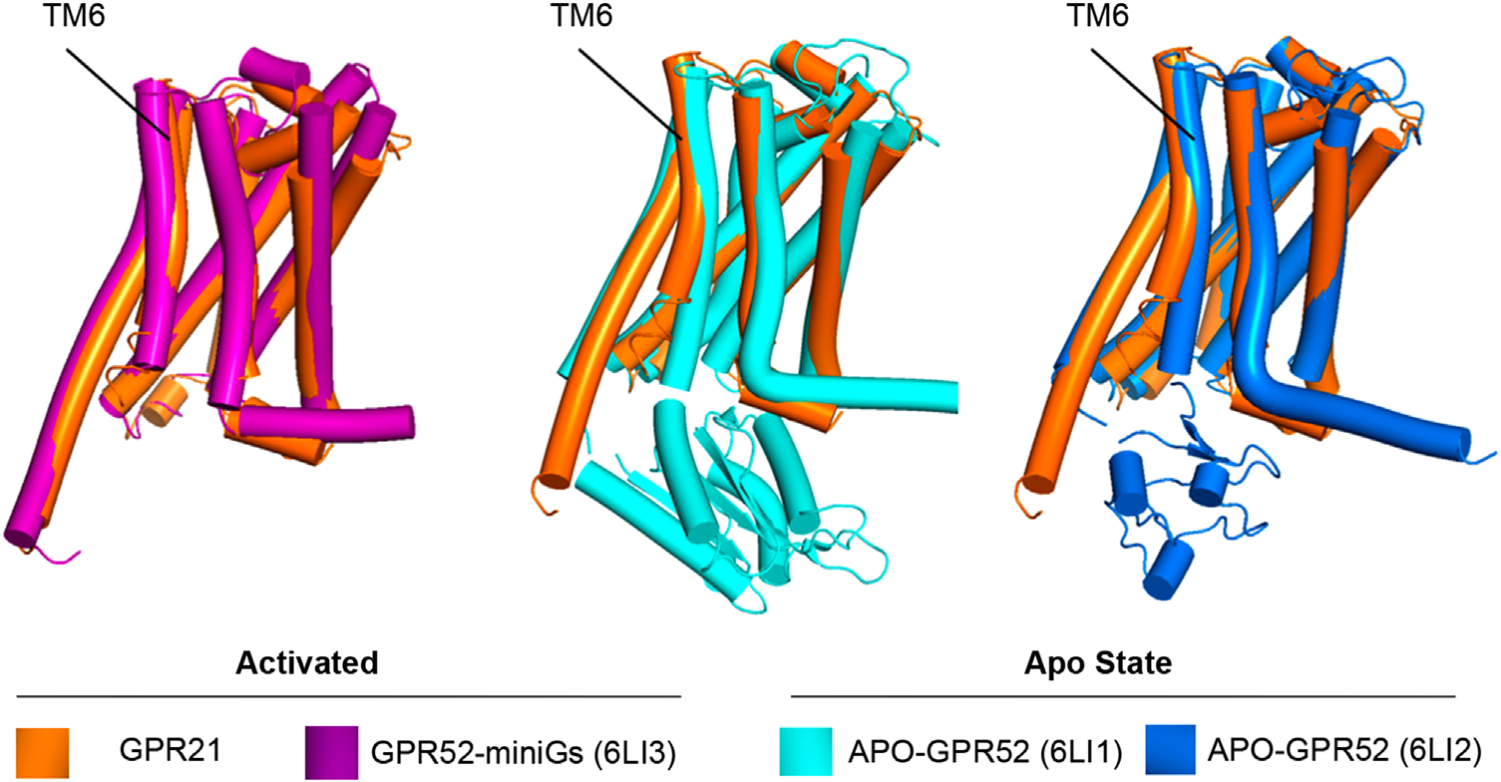
Structure comparisons of active GPR21 with apo‐GPR52 and active GPR52. The structural alignment showing outward bending of TM6 of activated GPR21 compared to apo‐GPR52. GPR21 TM6 conformation showed high consistency compared to miniGs‐coupled GPR52.

As GPR21 exhibited high structural similarity with GPR52 (71% overall sequence identity), we first compared the Gs‐coupled GPR21 with apo‐GPR52 structures (PDB: 6LI1 and 6LI2) and mini‐Gs‐coupled GPR52 (PDB: 6LI3).^14^ The ligand‐free apo‐GPR52 structures were in an inactive conformation in the absence of intracellular G protein. Our GPR21 structure exhibited high conformation similarity to the mini‐Gs‐coupled GPR52 Cα root mean squared deviation (r.m.s.d.): 1.032 Å. Gαs‐coupled GPR21 showed prominent outward displacement of transmembrane helix 6 (TM6), the hallmark feature of class A GPCRs (Figure 2). The backbone conformation of GPR21 TM6 is nearly identical to mini‐Gs‐coupled GPR52 (Figure 2). The data suggested that the GPR21 structure represented an active state GPCR.

### 2.3 Microswitches activation in GPR21

**FIGURE 3:**
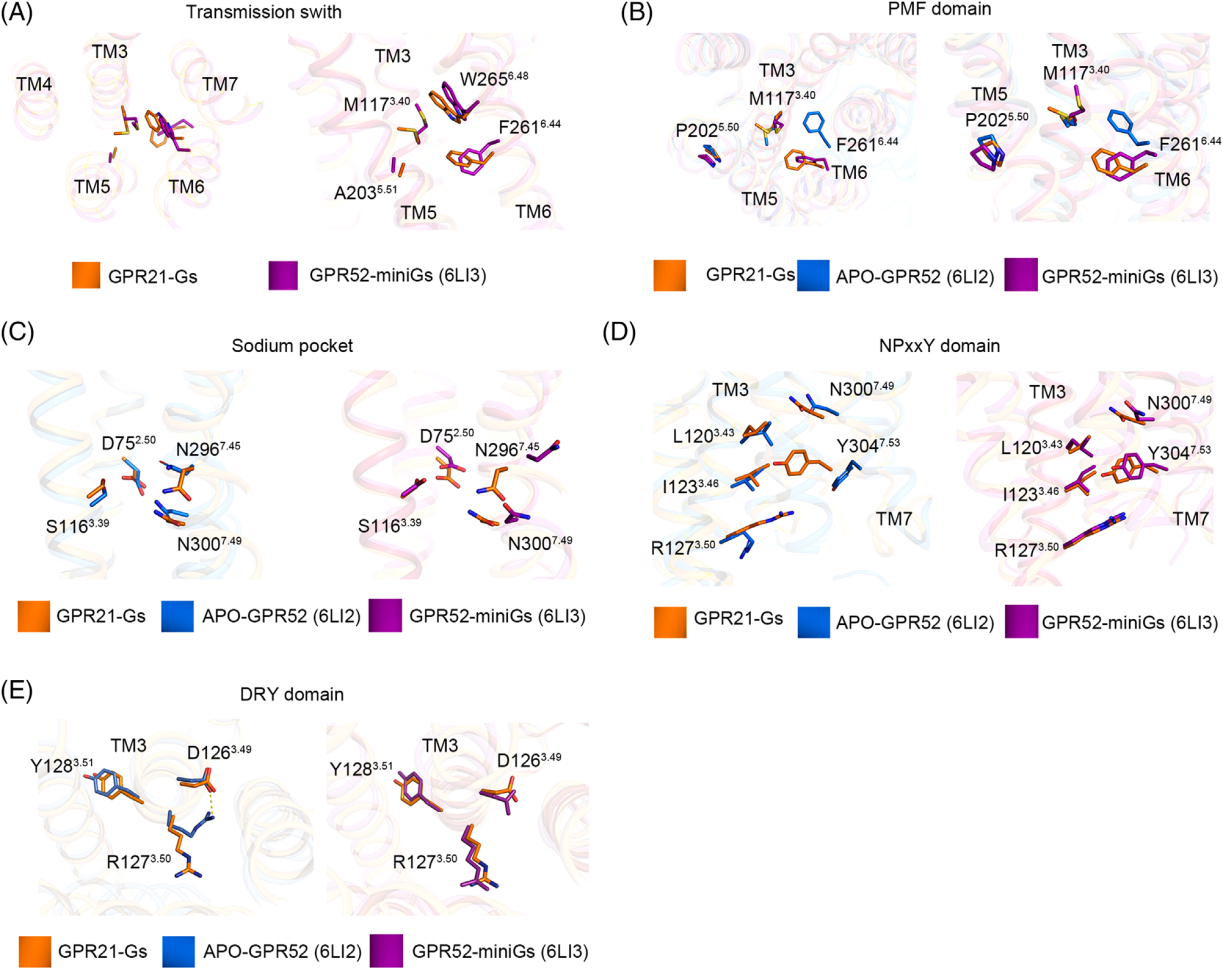
Comparison of conserved motifs of active GPR21, apo‐GPR52 and active GPR52. Comparison of transmission switch (A), PMF domain (B), sodium pocket (C), NPxxY domain (D), and DRY domain (E) indicated that GPR21 was in an active form.

In ligand‐activated class A GPCRs, ligand binding could induce a rotameric switch of W^6.48^ in the CWxP motif on TM6. The classical W^6.48^ rotation was also observed when GPR52 transited from inactive to active state. The side chains of F^6.44^ (in the PMF domain) and W^6.48^ rotated to face TM3, which brought TM6 closer to TM3 and increased intrahelical contract between transmission switches in the active conformation (Figure 3A,B). In β2AR, repacking of Na^+^ pocket residues triggered TM6 to shift toward TM4 when the receptor transited from inactive to active (PDB: 3SN6) state. In GPR21, however, the Na^+^ pocket residues showed high conformation consistency compared to the inactive GPR52 structure (Figure 3C). The rotameric switch of Y^7.53^ in the NPxxY domain brought GPR21 and GPR52 TM7 closer to TM3, which generated a new contact interface between TM7 and TM3 (Figure 3D). In the inactive GPR52, the salt bridge between residues D^3.49^ and R^3.50^ in the DRY domain maintained the structure in an inactive state. Similar to the active GPR52, GPR21 exhibited a rotameric switch on R^3.50^, which opened the ionic lock to facilitate Gαs‐coupling (Figure 3E).

### 2.4 G protein‐coupling GPCR21

**FIGURE 4:**
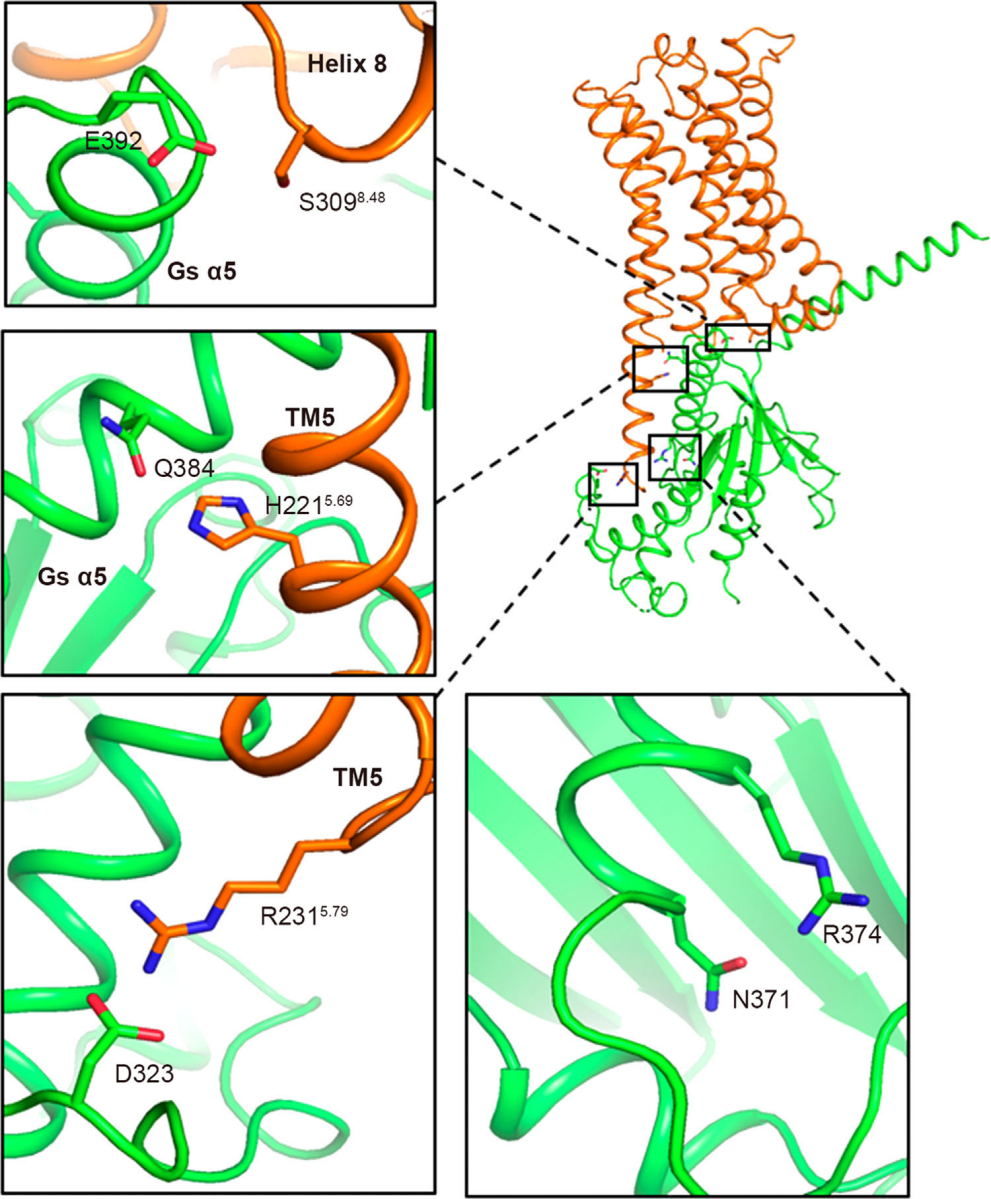
A close‐up view showing residues composing GPR21‐Gs interface. Interaction between GPR21 and α5 helix of Gαs is presented. Key residues are shown as sticks.

The intracellular milieu of TM5 and TM7 formed the contact interface on GPR21 for Gαs. TM5 is the longest transmembrane helix in GPR21. Polar interactions between TM5/7 and α5 helix of Gαs stabilized the GPR21‐Gαs complex and helped to maintain the active conformation. Further, the conformation and orientation of the α5 helix are strengthened by a hydrogen bond between two closely located residue pairs (N371 and R374) on Gαs (Figure 4).

### 2.5 GPR21 ECL2 occupied the orthosteric ligand‐binding pocket

**FIGURE 5:**
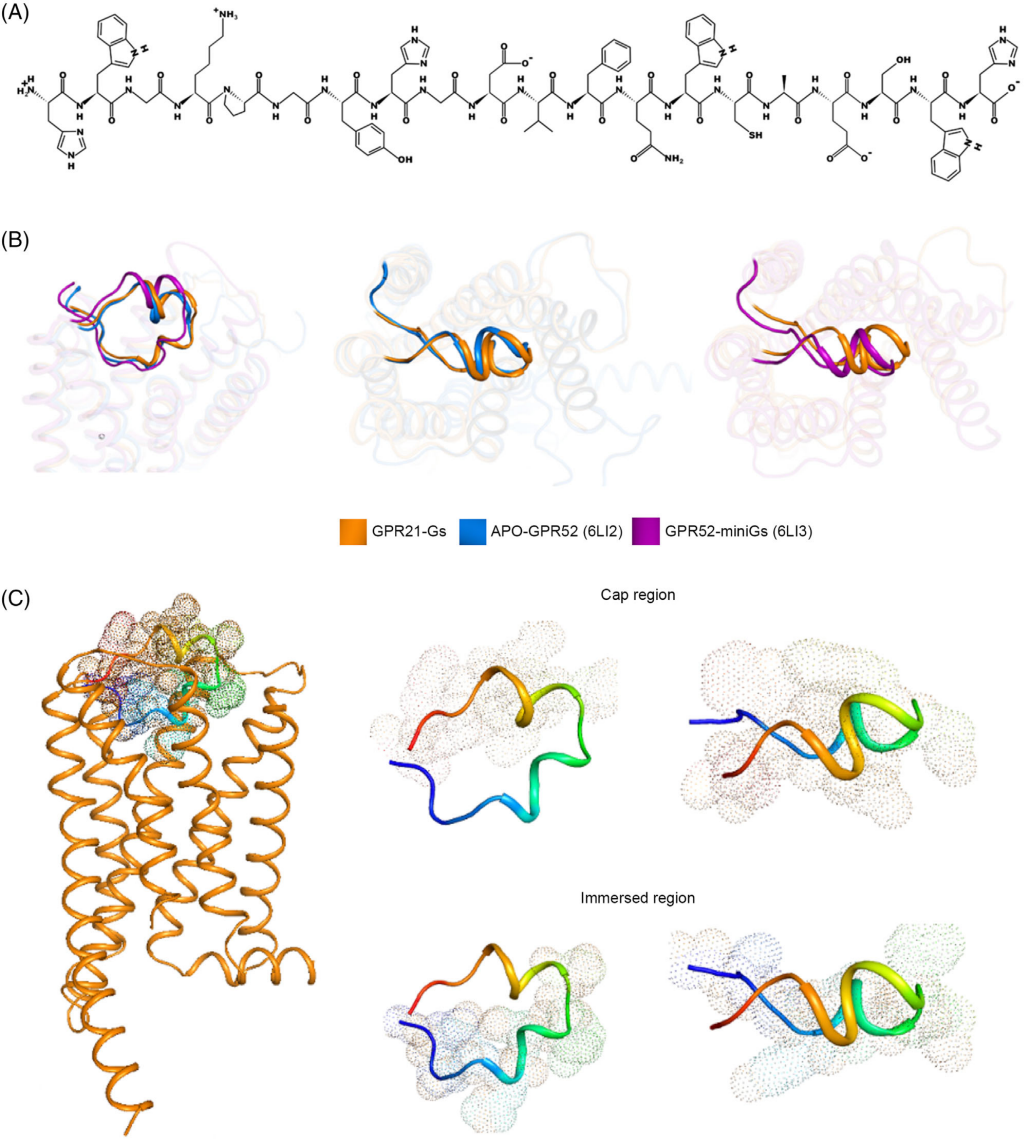
ECL2 of GPR21 occupied orthosteric ligand‐binding pocket and mediated the activation of GPR21. (A) Amino acid residues of GPR21 ECL2. (B) Structure superimposition of ECL2 from Gs‐coupled GPR21, mini‐Gs‐coupled GPR52 and apo‐GPR52. ECL2 occupied similar orthosteric ligand‐binding pocket. (C) A cartoon representation of GPR21 ECL2. GPR21 ECL2 is composed of two different regions: immersed region (169–178) and the cap region (179–188).

GPR21 ECL2 connected TM4 and TM5 at the extracellular side. The GPR21 ECL2 was intermingled with amino acids containing bulky/long side chains, which occupy the orthosteric ligand binding pocket (Figure 5A). ECL2 of GPR21 exhibited high conformational consistency compared to the apo‐GPR52 and Gs‐coupled GPR52 structures (Figure 5B). Structurally, ECL2 was composed of two different regions: immersed region and the cap region (Figure 5C).

### 2.6 ECL2‐induced GPR21 activation

**FIGURE 6:**
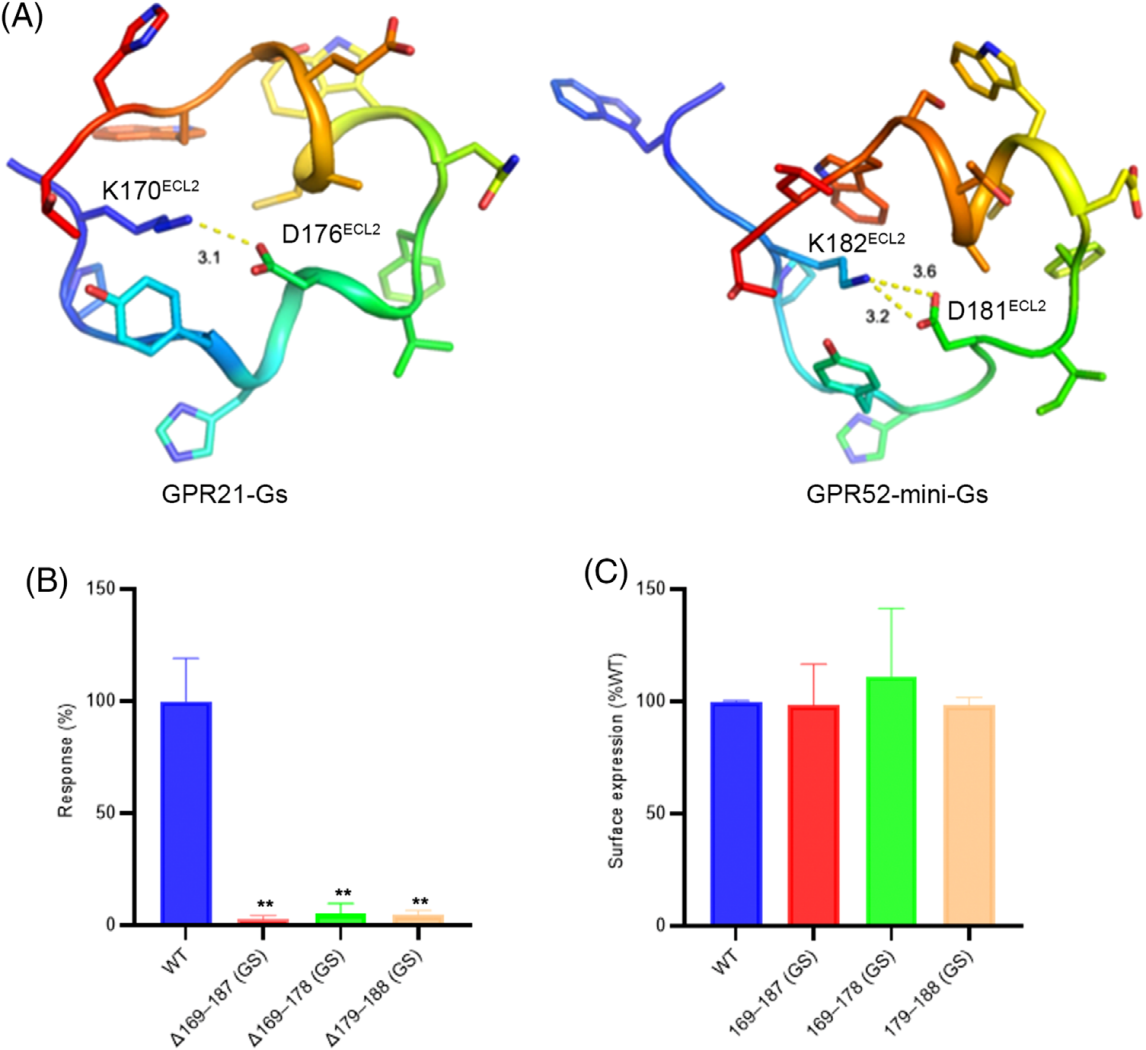
Mutations that influence the conformation of the ECL2‐abrogated downstream signaling of GPR21. (A) Intra‐loop ionic lock stabilizing GPR21/52 ECL2 in G protein‐coupled receptors. Key residues are shown as sticks. (B) Mutations that influence the conformation of the ECL2‐abrogated cAMP accumulation. The cAMP response level in Δ169–187 (GS), Δ169–178 (GS), and Δ179–188 (GS) was compared with that of WT. **p < 0.01, *t*‐test, *n* = 3. (C) Flow cytometry analysis of surface expression level of WT GPR21, Δ169–187 (GS), Δ169–178 (GS), and Δ179–188 (GS)

ECL2 started with a loop that plunged into the TM bundle's hydrophobic core, forming a submerged structure surrounded by residues from TM3 to TM7. The immersed region was constrained in position via an intra‐loop salt bridge (K170^ECL2^ and D176^ECL2^). As a result, residues between the salt bridge (P171^ECL2^, G172^ECL2^, Y173^ECL2^, H174^ECL2^, G175^ECL2^) are tethered to dip into the pocket, which brought ECL2 closer to TM3 (Figure 6A). Notably, the intra‐loop salt bridge in ECL2 was also present in the activated GPR52‐miniGs structure. The rest of the ECL2 formed a helical lid (cap region), protecting the immersed ECL2 loop (Figure 5C). Unlike other class A GPCRs, which might require agonist binding to induce a compact structure at the extracellular milieu, the cap region of GPR21 ECL2 was closed and anchored at the cognate ligand pocket with high stability. Consequently, GPR21 ECL2 was submerged and locked at the classical orthosteric pocket, which may prevent egress or access of ligands. As high GPR21 activity was observed in human tissues, we speculated that GPR21 ECL2 might promote the activation of GPR21 in ligand‐free conditions. Replacing ECL2 (cap region or immersed region) with a flexible GS linker significantly abrogated the downstream cAMP accumulation mediated by Gαs protein (Figure 6B,C). The results supported that GPR21 ECL2 was directly involved in the constitutive activation of GPR21 signaling.

## 3 DISCUSSION

Diabetes is a global health problem that burdens the healthcare systems. Over 90% of diabetes is type 2 diabetes (T2DM). The prevalence of diabetes has increased significantly in many countries.^15^ It is projected that the increasing trend for the global type 2 diabetes burden will continue. T2DM is a chronic disease resulting from chronic metabolic imbalance. At present, early diagnosis of T2DM remains difficult. T2DM diagnosis is based on plasma glucose criteria, including fasting plasma glucose levels, oral glucose tolerance test (OGTT), symptoms of hyperglycemia, and hemoglobin A1c (HbA1c) level.^16^ Patients with T2DM are at high risk of developing short‐term and long‐term complications, including cardiovascular disease (CVD), microvascular diseases (e.g., retinopathy, diabetic nephropathy, and neuropathy), and macrovascular diseases in arteries affecting the blood supply to different parts of the body. Further, T2DM patients are susceptible to other diseases, such as chronic liver and kidney disease, ischemic stroke, and cancers.^17^, ^18^ Cardiovascular disease is the leading cause of death and disability in T2DM, accounting for 10% of the fatal cases.^19^ Etiological factors, such as age, gender, lifestyle, dietary patterns, socioeconomic status, and genetic factors, are closely linked to the development of T2DM.^15^, ^20^, ^21^, ^22^

Compared to type 1 diabetes (T1DM), which is featured by insufficient insulin production by the pancreas, pancreatic β‐cell dysfunction, and insulin resistance are hallmark features of T2DM patients. Reduced β‐cell mass in T2DM patients will promote glucagon release from α‐cells contributing to reduced insulin secretion in T2DM. Further, insulin‐sensitive tissues will reduce glucose uptake by phosphorylating insulin receptor substrate (IRS)‐1 via serine/threonine kinase cascade, leading to insulin resistance in T2DM.^23^ Apart from the progressive hyperglycemic environment, dietary fat promotes IRS‐2 phosphorylation in the nonalcoholic fatty liver.^23^ T1DM can be treated by continued insulin injection. In contrast, combinatory treatment based on specific causes or symptoms is required to successfully control blood glucose in T2DM. Hence, identifying new treatment targets for drug development is beneficial.

Obesity (central visceral adiposity) is an epidemiological risk factor associated with the development of T2DM.^24^ Obesity is a prominent risk factor for T2DM in children and adolescents. Apart from storing excess nutrients, adipose tissue is regarded as an immune organ due to the large variety of adipose‐resident immune cell populations. Thus, accumulating adipose tissues will enhance proinflammatory cytokine release, gradually leading to chronic low‐grade systemic inflammation.^25^ It is noted that proinflammatory cytokine release from adipocytes is associated with obesity‐related inflammation insulin resistance. For instance, tumor necrosis factor‐alpha (TNF‐alpha), the primary inflammatory factor produced by immune cells, can interfere with early insulin‐stimulated tyrosine phosphorylation of insulin receptors.^26^ It is suggested that TNF‐alpha is the linkage between insulin resistance and beta‐cell dysfunction by altering glucose homeostasis in the liver and pancreas.^27^ Proinflammatory cytokine can promote lipolysis and free fatty acids release from adipocytes. The elevated circulating acids stimulate hepatic glucose production contributing to persistent hyperglycemic conditions. Currently, treatment targeting obesity‐associated T2DM by anti‐inflammatory agents is less well appreciated. Given that the cause of T2DM is multifactorial, a combination treatment with different antidiabetic agents is required. Therefore, identifying new treatment targets and continued development of new medication is essential for treating T2DM.^28^

GPCR is one of the promising treatment targets for T2DM.^29^ More than 30 GPCRs had been reported to be associated with the pathophysiologic development of T2DM. Riddy et al. reviewed the involvement of GPCR in developing (a) β‐cell dysfunction/insulin resistance T2DM, and (b) obesity‐induced T2DM.^29^ Most of the reported GPCRs are associated with the metabolic dysfunction of β‐cell.^29^ Examples included adrenoceptors, cannabinoid receptors, prostaglandin E2, free fatty acid receptors, G protein‐coupled estrogen receptor (GPER), and melatonin receptors. For example, drugs targeting glucagon‐like peptide 1 receptor (GLP‐1R) agonist are approved for T2DM treatment. In the case of T2DM caused by β‐cell dysfunction or insulin resistance, synthetic agonist (glucagon‐like peptide GLP) targeting glucagon‐like peptide 1 receptor (GLP1R) showed promising therapeutic effects.^30^ GLP1R is class B1 (secretin) GPCR widely expressed in the pancreas, lungs, and central nervous system. Activated GLP1R can stimulate insulin release by stimulating cAMP accumulation and promoting IP3‐mediated Ca^2+^ release. The synthetic GLP1R agonist has a longer half‐life than the endogenous GLP. Orphan GPR119 is suggested to be a novel target for T2DM resulting from β‐cell dysfunction and insulin resistance.^2^ For obesity‐induced T2DM, several class A orphan GPCRs, including GPR21, GPR35, GPR84, and GPR132, are suggested to play an active role in the developmental processes of T2DM.^29^

Activated GPCRs could couple preferentially with different Gα proteins (Gαs, Gαi/o, Gαq/11, Gα12/13) to trigger a downstream signal transduction cascade. Gα protein subtypes regulate the generation of different secondary messengers and trigger a different downstream signaling cascade. It is established that GPR21 is implicated in type 2 diabetes by Gαq signaling pathways. Bresnick et al. (2003) reported that orphan GPR21 is coupled to Gαq in their in‐house transcription‐based reporter assay, designed for elucidating downstream signal transduction pathways for orphan GPCRs.^31^ Upon activation, Gαq‐biased GPCR will recruit Gq/11 subfamily of G proteins. Gαq protein‐coupled GPCRs are frequently found in chemokine and hormone receptors associated with immunity and autoimmune disease.^32^ The activated Gαq protein simulates the phospholipase C (PLC) isozymes to hydrolyze membrane phospholipids PIP2 (phosphatidylinositol 4,5‐bisphosphate) to generate the second messenger inositol 1,4,5‐triphosphate (IP3) and diacylglycerol (DAG). IP3 is a Ca^2+^‐mobilizing second messenger. IP3 could bind to the ion channels on ER and promote calcium ion release. DAG is a protein kinase‐activating molecule. DAG and calcium ions activate protein kinase C and trigger a cellular response by phosphorylating the target protein.

In a recent functional study on GPR21 expression in macrophages, the authors measured inositol‐1‐phosphate 1 (IP1) changes in the presence of GRA2 (2‐(1‐naphthyloxy)‐N(2‐phenoxyphenyl)acetamide), the inverse agonist of GPR21. Dose‐dependent reduction of IP1 in macrophage‐like cells supported the existence of constitutive Gαq/11 signaling in GPR21 cells.^33^ Gαq/11 can activate phosphoinositide 3‐kinase (PI3K)/AKT signaling cascade in T2DM. PI3K/AKT regulates glucose and lipid metabolism in insulin‐sensitive tissues, such as skeletal muscle, adipose tissues, liver, pancreas, and brain.^34^ In obese mice and rhesus monkeys models, targeting PI3K is a safe approach to reduce bodyweight, adiposity and lower serum glucose levels.^35^ Therefore, pharmacologically inhibiting the Gαq‐coupling receptor might be an effective approach for T2DM treatment.

GPR21 remains listed in the class A orphan GPCR with no validated endogenous ligands. Kakarala et al. attempted to rule out the potential ligands for GPR21 using sequence structure‐based phylogenetic classification on class A GPCRs. Maximum likelihood and evolutionary analysis demonstrated that GPR21 and its closest homolog GPR52 formed a distinct cluster with no association with other class A rhodopsin receptors. Their results suggested that GPR21/GPR52 are likely free fatty acids or leukotrienes receptors.^36^ At present, the speculation is not supported by experimental data. Although circulating free fatty acids are closely associated with hepatic glucose release, there is still no evidence supporting GPR21 mediates D2TM pathogenesis by responding to the fatty acid signals.

In a study measuring the constitutive activity among orphan class A GPCR, GPR21 and GPR52 exhibit constitutive signaling functions in CHO‐K1 wild‐type Chinese hamster ovary cells without specific ligands.^37^ Phenotypically, Gpr21 knockout mice exhibited a leaner and more active phenotype with decreased chemoattractant protein MCP‐1 (CCL2) levels.^38^ Bordano et al. demonstrated that GPR21 expression is remarkably increased in THP‐1‐derived M1 macrophages. GPR21 gene knockdown experiment on both M1 macrophages deceased TNF‐α and IL1‐β production.^33^ In white adipose tissue (WAT) and immune cells of DT2M patients, parallel upregulation of GPR21 and CCL2 was observed.^33^ Together, the data suggested that GPR21 might mediate chronic, low‐grade inflammatory response by modulating proinflammatory cytokine release and migration of immune cells.

Although GPR21 is reported to be a Gαq‐coupling receptor, the clear electron density map of our GPR21‐Gαs structure provides direct evidence supporting that GPR21 could also be a Gs‐coupling GPCR. Current data suggested that GPR21 could remain active in physiological or pathological conditions without any ligands. After dissociating from the activated GPCR, Gαs could stimulate cAMP generation through activating adenylyl cyclase. Subsequent binding of cAMP on PKA could phosphorylate specific cytoplasmic targets with different outcomes depending on cellular context. Gαs‐mediated adenylyl cyclase activity increase is frequently reported in diabetes. Given that we could not obtain the stable Gαq‐coupling receptor, we could about deduce the coupling preference (Gαs over Gαq) from the structure perspective. As Gαq signaling mediated by GPR21 has been noted in macrophages recently, the preferential G protein coupling effects might be dependent on cellular context.^33^ Changes in residue contacts in the Na^+^ pocket are an important initial step for ligand‐induced class A GPCR activation.^4^ In the GPR21 structure, we noted high conformation consistency compared to the inactive GPR52 structure. As cAMP signaling remains active in the GPR21, the receptor activation function could be shared by the disconnected motif PMF, which is spatially close to the Na^+^ pocket.

Targeting the cAMP/PKA pathway is suggested to be helpful in glycemic control.^9^ In pancreatic islets, cAMP/PKA pathways control glucagon levels in circulation through activating transcription of the glucagon gene.^39^ PKA‐dependent GLUT2 phosphorylation at the carboxyl‐terminal tail reduced the glucose uptake efficacy of pancreatic beta cells.^11^ In the liver, PKA could facilitate gluconeogenesis or inhibit glycogen synthase activity depending on the energy state.^39^ In addition, the activated cAMP/PKA signaling cascade will affect the response of diabetic patients to metformin, the drug for regulating glucose levels in type 2 diabetes. He et al. demonstrated that metformin resistance/insensitivity is partly caused by cAMP/PKA activation, which phosphorylates AMP‐activated protein kinase (AMPK) in hepatocytes.^40^ Hence, the functional impact of GPR21‐mediated Gαs activation in type 2 diabetes warrants further investigation.

All class A GPCR has a common structure with seven transmembrane helices linked by alternating extracellular loops (ECLs) and intracellular loops (ICLs). Structure data established that the highly flexible ECL is involved in ligand recognition and receptor activation.^13^ ECL2 is essential for class A GPCR to select an accurate ligand in receptor transduction and signal activation. Further, ECL2 allows members of the same subtype to distinguish ligands with different chemical compositions. In most reported GPCR structures, the density of ECLs is poor or missing due to technical constraints in crystallographic data interpretation and single particle analysis. The 3D coordinate of class A GPCR ECL2 is hard to be modeled by computational prediction approaches as the extracellular loop exhibits diverse conformation differences.^41^, ^42^

GPR52 is the only homolog of GPR21. Around 70% of their sequences are identical. Both of them are orphan class A GPCR with no characterized endogenous ligands. GPR21 and GPR52 could maintain active receptor conformation without agonists. Similar to GPR21, high basal activity of GPR52 is observed. GPR25 is also a Gs‐coupling receptor. Generally, GPCRs are ligand‐activated receptors. Upon ligand engagement in the orthosteric ligand‐binding pocket, the receptor adopts an active conformation to accommodate Gαs binding. In the case of GPR21/52, ECL2 functions as a built‐in ligand for receptor self‐activation. GPR21 ECL2 is highly similar to GPR52 ECL2. The solid density map in cryo‐EM analysis suggested that GPR21 and GPR52 ECL2s are inherently stable structures. The cap region and pocket region of GPR21 ECL2 contain helical structures, which help maintain ECL2 conformation in the GPR21 pocket. From the GPR52 structure, we noted that the ECL2 conformation is highly stable and did not demonstrate apparent displacement or rearrangement when the receptor transited from inactive to an active form.

As ECL2 occupied most of the orthosteric pocket and left little room for diffusible ligands, it is speculated that synthetic modulators targeting the cognate ligand binding sites of class A GPCRs will have limited potency. As shown in the study of Lin et al., the synthetic allosteric ligand for GPR52 has to be small with high shape/size accuracy to fit into the shrinking side pocket of GPR52. Although ECL2 is an effective self‐modulator on GPR21/52 activity, it has been shown that therapeutic modulation via the remote allosteric site is still a feasible approach in treating Huntington's disease and psychiatric disorders.^43^, ^44^ The atomic GPR21 structure presented in this study might provide an essential fundamental basis for the structural‐based drug development process.

## 4 MATERIALS AND METHODS

### 4.1 Constructs of GPR21, DNGαs, Gβ1, and Gγ2

The human GPR21 was synthesized by GENERAL BIOL (Chuzhou, China) and cloned into the pFastbac1 vector (Gibco), harboring an N‐terminal Flag tag. The expression of GPR21 was low in Sf9 insect cells. Our previous experiences showed that a BRIL fusion significantly improved the expression of class A GPCR. Therefore, BRIL was inserted in the N‐terminal of GPR21 to enhance the expression of GPR21. A dominant‐negative human Gαs (DNGαs) was cloned into the pFastbac1 vector (Gibco). Gβ1 and Gγ2 were cloned into the pFastBac‐Dual vector (Gibco).

### 4.2 Expression and purification of GPR21‐DNGαs complex

Bac‐to‐Bac system was employed to express the GPR21‐DNGαs complex in Sf9 insect cells. In brief, the BRIL‐GPR21 construct was transformed into DH10Bac to produce the recombinant bacmid. Polymerase chain reaction (PCR) was performed to confirm the insertion of BRIL‐GPR21 into bacmid. Sf9 cells were transfected with bacmid to obtain P1 baculovirus using FuGENE HD Transfection Reagent (Promega). Then, P1 baculovirus was used to infect Sf9 cells to obtain P2 baculovirus. GPR21, DNGαs, Gβ1, and Gγ2 were co‐expressed in Sf9 cells. The cells were harvested 48 h after infection of P2 baculovirus. The cell pellet was lysed in lysis buffer containing 10 mM HEPES pH 7.5, 1 mM EDTA, and 4 mg/ml iodoacetamide, followed by centrifugation to obtain cell membranes. Then, solubilization buffer containing 20 mM HEPES pH 7.5, 100 mM NaCl, 1% lauryl maltose neopentyl glycol (LMNG), 0.1% cholesteryl hemisuccinate (CHS), 10% glycerol, apyrase, 10 mM MgCl_2_, 4 mg/ml iodoacetamide, 2.5 μg/ml leupeptin, and 0.16 mg/ml benzamidine were added to solubilize the cell membranes. The lysate was incubated with anti‐FLAG M1 affinity resin for 1 h at 4°C in the presence of 2 mM calcium chloride. After centrifugation at 2500 rpm for 5 min at 4°C, the resin was washed extensively with wash buffer containing 20 mM HEPES pH 7.5, 100 mM NaCl, 0.003% LMNG, and 2 mM calcium chloride. The protein complex was eluted with 20 mM HEPES pH 7.5, 100 mM NaCl, 0.003% LMNG, 0.001% GDN, 0.004% CHS, 200 μM flag peptide, and 5 mM EDTA. The elution fractions were incubated with Nanobody‐35 (Nb35) and subjected to size‐exclusion chromatography on a Superose 6 increase 10/300 column (GE) with a running buffer containing 20 mM HEPES pH 7.5, 100 mM NaCl, 0.003% LMNG, 0.001% GDN, and 0.004% CHS. The peak fractions containing GPR21‐DNGαs complex were concentrated to 5 mg/ml and subjected to cryo‐EM sample preparation.

### 4.3 Expression and purification of Nb35

Nb35 was cloned into the pET28a vector and expressed in *Escherichia coli* strain BL21 (DE3). The cells were cultured in TB medium supplemented with 0.1% glucose, 1 mM MgCl_2_. When OD_600_ reached 0.6, 1 mM isopropyl β‐D‐thiogalactoside (IPTG) was added to the culture to induce Nb35 expression, followed by the culture at 18°C for 24 h. The cells were collected by centrifugation and lysed in TES buffer containing 0.2 M Tris pH 7.5, 0.5 mM EDTA, and 0.5 M sucrose at 4°C for 1 h. Then, an equal value of H_2_O was added, and the cells were incubated at 4°C for 45 min. After centrifugation, the supernatant was incubated with nickel resin at 4°C for 1 h. The nickel resin was washed with washing buffer 1 containing 20 mM HEPES pH 7.5, 500 mM NaCl, and 20 mM imidazole, followed by wash buffer 2 containing 20 mM HEPES pH 7.5, 100 mM NaCl, and 20 mM imidazole. The NB35 protein was eluted with 20 mM HEPES pH 7.5, 100 mM NaCl, and 300 mM imidazole. The elution fractions were loaded onto a Superose 6 increase 10/300 size exclusion column (GE) with a running buffer containing 20 mM HEPES pH 7.5 and 100 mM NaCl. Peak fractions were harvested and concentrated to 5 mg/ml. The purified Nb35 protein was supplemented with 10% glycerol and stored at −80°C.

### 4.4 Cryo‐EM sample preparation and data acquisition

The GPR21‐DNGαs‐Nb35 complex was loaded onto glow‐discharged holey carbon grids (Quantifoil R1.2/1.3). The grids were blotted for 4 s to remove excess samples and were subsequently plunged into liquid ethane using Vitrobot Mark IV (Thermo Fisher Scientific). Cryo‐EM images were obtained on a 300 kV Titan Krios Gi3 microscope at the Kobilka Cryo‐EM Center of the Chinese University of Hong Kong (Shenzhen). The pixel size is 0.83 Å. SerialEM5 software was employed to record micrographs.

### 4.5 Cryo‐EM data processing

Data processing was performed using CryoSPARC. In total, 6443 movies were subjected to motion correction and CTF estimation, resulting in 6006 micrographs for further processing. The micrographs were subsequently subjected to 2D classification, producing 393,876 particles for 3D refinement. The final refinement generated a map with an indicated global resolution of 2.91 Å (Figure 1).

### 4.6 Model building and refinement

AlphaFold was used to generate the initial model for human GPR21. The corticotropin‐releasing factor 1 receptor‐Gs‐Nb35 complex (PDB 6PB0) was employed to produce the initial models for Gs and Nb35. UCSF chimera was used to dock all the initial models into the EM density map. Then, COOT was employed to adjust and rebuild models, followed by real‐space refinement using Phenix. The final refinement statistics were validated using Molprobity and are shown in Table 1.

**TABLE 1:** Cryo‐EM data collection, refinement, and validation statistics

**Data collection and processing**	**GPR21‐Gs‐Nb35**
Magnification	105,000
Voltage (kV)	300
Electron exposure (e–/Å)	45.51
Defocus range (μm)	−1.0 to −2.0
Pixel size (Å)	0.85
Symmetry imposed	C1
Initial particle images (no.)	393,876
Final particle images (no.)	149,012
Map resolution (Å)	2.91
FSC threshold	0.143
**Refinement**	
Model resolution (Å)	2.91
Map sharpening B factor (Å)	−89.6
**Model composition**	
Nonhydrogen atoms	8067
Protein residues	1015
**B factors (Å)**	
Protein	57.74
**r.m.s. deviations**	
Bond lengths (Å)	0.002
Bond angles (°)	0.439
**Validation**	
MolProbity score	1.2
Clashscore	4.12
**Ramachandran plot**	
Favored (%)	98.29
Allowed (%)	1.71
Disallowed (%)	0

### 4.7 cAMP accumulation assay

To investigate the role of ECL2 in the activation of GPR21, three GPR21 mutants were generated. The residues 169–178 (immersed region of ECL2), residues 179–188 (cap region of ECL2), and residues 169–187 (the whole ECL2) were replaced by a flexible GS linker (GGSGGS), respectively. Wild‐type GPR21 and GPR21 mutants were cloned into pCDNA3.1 vector and expressed in HEK293 cells (Invitrogen). Plasmids were transfected in HEK293 cells using lipofectamine 3000 (Invitrogen). cAMP accumulation was performed using HTRF cAMP kit (Cisbio Bioassays) following the manufacturer's instructions. In brief, HEK293 cells were seeded into 384‐well plates. Then, 5 μl of cAMP‐d2 reagent and 5 μl of cAMP Eu‐cryptate antibody were added to each well. After incubation at room temperature for 1 h, fluorescence was measured using a microplate reader (Envision 2105, PerkinElmer) with excitation at 330 nm and emission at 620 and 665 nm. cAMP accumulation was calculated from a standard dose–response curve using GraphPad Prism 8.0 (GraphPad Software). The cAMP levels of GPR21 mutants were normalized to that of wild‐type GPR21 (100% level).

### 4.8 Flow cytometry analysis

HEK293 cells transfected with wild‐type GPR21 and GPR21 mutants were harvested and incubated with phosphate buffer solution (PBS) plus 0.5% bovine serum albumin (BSA) for 15 min, followed by incubation with anti‐FLAG antibody (Thermo Fisher) at 4°C for 1 h. After washing with PBS three times, the cells were incubated with Alexa‐488‐conjugated secondary antibody (Beyotime) at 4°C for 1 h. After washing with PBS three times, the cells were subjected to flow cytometric analysis on an AccuriTM C6 Plus flow cytometer (BD). FL1 channel was employed to record fluorescent signal derived from Alexa‐488. The surface expression levels of GPR21 mutants were normalized to that of wild‐type GPR21 (100% level).

## AUTHOR CONTRIBUTIONS

Thian‐Sze Wong, Wei Gao, Geng Chen, Chen Qiu, and Guodong He performed the experiment, data collection, model building, and manuscript preparation. Fang Ye, Zhangsong Wu, and Zicheng Zeng contributed to the writing and figure/table preparation. Thian‐Sze Wong and Yang Du supervised the project and revised the manuscript. All authors contributed to the article. All authors have read and approved the final version of the manuscript

## CONFLICT OF INTEREST

The authors declare they have no conflicts of interest.

## ETHICS APPROVAL

Not applicable.

## Data Availability

The data supporting this study are available from the corresponding author upon reasonable request. The cryo‐EM density map and structure coordinate of GPR21‐Gs complex have been deposited to the Protein Data Bank database (PDB) and Electron Microscopy Database (EMDB) under accession codes 8HMV and EMD‐34903, respectively.
